# Decoding visual evoked potential latency: revealing neurological connections in Parkinson's disease

**DOI:** 10.25122/jml-2024-0319

**Published:** 2024-06

**Authors:** Diana Sipos-Lascu, Ştefan Cristian Vesa, Nicu-Catalin Draghici, Livia Livint Popa, Lacramioara Perju-Dumbrava

**Affiliations:** 1 Department of Neurosciences, Iuliu Hatieganu University of Medicine and Pharmacy, Cluj-Napoca, Romania; 2 Department of Pharmacology, Toxicology and Clinical Pharmacology, Iuliu Hatieganu University of Medicine and Pharmacy, Cluj-Napoca, Romania; 3 RoNeuro Institute for Neurological Research and Diagnostic, Cluj-Napoca, Romania; 4 IMOGEN Institute, Emergency Clinical County Hospital Cluj, Cluj-Napoca, Romania; 5 Neurology Clinic, Cluj-Napoca Emergency Clinical County Hospital, Cluj-Napoca, Romania

**Keywords:** Parkinson's disease, non-motor symptoms, apathy, anhedonia, visual evoked potentials

## Abstract

Parkinson's disease (PD) is a complex neurodegenerative disorder characterized by diverse motor and non-motor symptoms. Visual evoked potentials (VEPs) provide valuable insights into the neurological changes in PD. This study examines VEP latency to explore potential connections between visual processing and PD progression, focusing on whether inter-eye latency differences are influenced by disease severity and symptomatology. A cross-sectional observational study was conducted with 59 PD patients at the Neurology I Clinic, Cluj-Napoca County Emergency Clinical Hospital, from October 2019 to October 2021. Patients underwent neurological and psychological evaluations, including VEP testing with a reversal pattern technique. P100 wave latency was assessed for both eyes, and associations with clinical indicators like Hoehn and Yahr stages, UPDRS scores, and non-motor symptoms were analyzed. VEP latencies for the right and left eyes were 108.7 ± 10.6 ms and 108.4 ± 9.7 ms, respectively, with no significant inter-eye differences (*P* = 0.8). UPDRS item 4 scores correlated significantly with both latencies (*P* = 0.003 for the left eye and *P* <0.001 for the right). Latency differences between eyes were shorter in patients with symmetrical parkinsonism compared to those with unilateral predominance. Age correlated weakly with P100 latency, and a weak correlation was found between anhedonia scores and right-eye latency. VEP latency is sensitive to PD motor severity, with shorter inter-eye latency differences in symmetrical parkinsonism, suggesting balanced dopaminergic dysfunction. VEP latency differences offer insights into neurophysiological changes in PD, reflecting dopaminergic dysfunction and its impact on visual processing. These findings support the potential of VEPs as diagnostic and prognostic tools in PD assessment.

## INTRODUCTION

Parkinson's disease (PD) remains a complex neurological condition characterized by a spectrum of motor and non-motor symptoms, challenging both patients and clinicians alike [1]. The intricate interplay between the disease's diverse manifestations and its underlying neural alterations continues to drive scientific curiosity. Among the neurophysiological methods, visual evoked potentials (VEP) emerge as a promising approach for unraveling the intricate neurological aspects of PD [2]. The significance of investigating VEP latency within PD lies in its potential as a neurophysiological window, offering insights into the underlying neural changes accompanying the disease progression. Understanding these neural foundations holds the promise of advancing both diagnostic and prognostic, thereby paving the path for more precise therapeutic interventions and a comprehensive grasp of the complex neural dynamics governing the manifestations of Parkinson's disease. Moreover, emerging evidence suggests potential associations between alterations in VEP and the cognitive spectrum observed in dementia and depression. Alterations in VEP parameters have shown associations with depressive symptoms in neurological conditions [3-5]. Our study aimed to explore VEP latency within the context of PD. Specifically, the main objective was to examine whether the latency of VEP recorded from the right eye aligns with that of the left eye and to explore whether there was differential elongation observed in the contralateral eye of the more affected hemibody due to parkinsonism, potentially showing prolonged latency in the left eye in cases with right parkinsonism predominance. Furthermore, our inquiry aimed to investigate whether the difference in VEP latency between the left and right eyes exhibits more pronounced variations in individuals with advanced PD, as indicated by higher Hoehn and Yahr stage and the Unified Parkinson's Disease Rating Scale (UPDRS) scores. Additionally, we aimed to discern if variations in VEP latencies between both eyes are more pronounced in individuals exhibiting heightened psycho-affective non-motor symptoms, specifically apathy measured by the Lille apathy rating scale (LARS), Dimensional Apathy Scale (DAS), and UPDRS I item 4 scales, as well as anhedonia assessed by the SHAPS scale. Through this exploration, the research aims to contribute to the broader understanding of Parkinson's disease, shedding light on potential neurological markers that might ultimately guide more effective interventions and management strategies.

## MATERIAL AND METHODS

The current study was a cross-sectional, observational study and enrolled 59 patients with PD who were admitted to the Neurology I Clinic, Cluj-Napoca County Emergency Clinical Hospital, from 1 October 2019 to 31 October 2021.The inclusion criteria were as follows: patients with PD, Hoehn and Yahr stage 1–3, and the provision of signed documentation providing informed consent for voluntary participation in the study. Patients with mourning reactions and those who did not sign the informed consent form for participating in the study and/or the agreement regarding personal data processing for research purposes were excluded. The study was approved by the ethics committee of the Iuliu Hatieganu University of Medicine and Pharmacy, Cluj-Napoca, Romania (registration number: 90/2021). The following data were collected: age, gender, environment, smoking, coffee consumption, history of dementia, depression, and neurological examinations were performed, each of which was followed by the calculation of the UPDRS score and placement on the Hoehn and Yahr scale [6,7]. All participants underwent psychological evaluation, including the Mini-Mental State Examination (MMSE) scale [8]. Psychiatric examinations were performed on patients for whom the psychological evaluations highlighted depressive elements, increased emotional reactivity, moderate/severe anxiety (according to the Leahy anxiety scale), or moderate/severe cognitive impairment (according to MMSE) [8,9]. The patients were rated using apathy scales (the Lille Apathy Rating Scale, Apathy Evaluation Scale, Dimensional Apathy Scale, and UPDRS part I item 4) and anhedonia scales (Snaith–Hamilton Pleasure Scale) [6,10-13]. Cut-off values determined in validation studies for these scales were used [6,10-13].

Volunteers underwent VEP testing using a Keypoint 4, Medtronic, Denmark; software: Keypoint v. 5.11- Alpine BioMed) through the 'Reversal Pattern' technique. The reversal rate was 2 Hz. The test was performed on each eye separately on each subject, with the other eye covered during the test. The latencies of P100 were recorded. The difference between the latencies of the P100 wave recorded in the two eyes was calculated by subtracting the smaller value from the larger value regardless of the eye. All our scores and neurophysiological tests were performed while patients with motor fluctuations were in the 'on' phase. Statistical analyses were performed using the MedCalc Statistical Software version 19.6.4 (MedCalc Software Ltd., Ostend, Belgium; https://www.medcalc.org). Quantitative data are expressed as mean and standard deviation (normally distributed according to the Shapiro–Wilk test). Qualitative data are expressed as frequencies and percentages. Comparisons between the two groups were performed using the Student's t-test. A one-way ANOVA was used to compare differences among the three groups. The correlations between quantitative variables were verified using Pearson correlation, while Spearman rank correlation was employed for assessing relationships between ordinal variables. A *P* value < 0.05 was considered statistically significant.

## RESULTS

The latency P100 for the right eye was 108.7 ± 10.6, and for the left eye, 108.4 ± 9.7 (*P* = 0.8). The latency P100 for the right eye and left eye, or the difference in latency between eyes, did not show statistically significant differences concerning demographic factors, personal habits, neuro-psychiatric diagnosis, or functional disability score (as shown in Table 1). The predominant type of parkinsonism significantly affected the difference in latency between the eyes. Patients with symmetrical parkinsonism displayed a shorter difference in latency between the eyes compared to patients with either right-sided (*P* = 0.01) or left-sided parkinsonism (*P* = 0.003).

**Table 1:** Variation of latency P100 for the right eye, left eye, and difference in latency between eyes regarding several parameters

Variables Values		Latency P100 for the right eye		Latency P100 for the left eye		Difference in latency between eyes	
		Values	*P*	Values	*P*	Values	*P*
Environment	Rural (*n* = 8)	105.6 ± 10.2	0.3^†^	105.1 ± 7.5	0.3^†^	2 (0.62; 4.5)	0.7^†^
	Urban (*n* = 51)	109.2 ± 10.7		108.9 ± 9.9		2 (1; 5)	
Gender	Male (*n* = 32)	110.6 ± 10.8	0.1^†^	109.8 ± 8.8	0.2^†^	2 (0.77; 5.3)	0.7^†^
	Female (*n* = 27)	106.4 ± 10.2		106.7 ± 10.5		2 (1; 4)	
Smoking	No (*n* = 41)	107.3 ± 9.3	0.1^†^	107.5 ± 9.8	0.3^†^	2 (1; 4)	0.3^†^
	Yes (*n* =18)	111.9 ± 12.4		110.2 ± 9.4		3.5 (0.92; 6.2)	
Coffee consumption	No (*n* = 15)	106.8 ± 7.7	0.4^†^	107 ± 8.5	0.5^†^	2 (1; 5)	0.8^†^
	Yes (*n* = 44)	109.3 ± 11.5		108.8 ± 10.1		2 (1; 4.3)	
Depression	No (*n* = 38)	110.2 ± 11.3	0.1^†^	109.6 ± 9.5	0.2^†^	3 (1; 6)	0.06^†^
	Yes (*n* = 21)	106 ± 8.9		106.2 ± 9.9		1 (0.6; 3.25)	
Dementia	No (*n* = 57)	109 ± 10.7	0.2^†^	108.5 ± 9.8	0.4^†^	2 (1; 5)	0.8^†^
	Yes (*n* = 2)	100.5 ± 3.5		103 ± 2.8		2.5 (2; -)	
Predominant side	Symmetric (*n* = 13)	109.2 ± 8.4	0.07^††^	179.6 ± 8	0.9^††^	1 (0.5; 1.75)	0.01^††^
	Right (*n* = 29)	105.8 ± 9.5		108.4 ± 10.9		2 (1; 4)	
	Left (*n* = 17)	113.2 ± 12.7		108.9 ± 9.2		5 (0.85; 7.6)	

† Student t test; †† one way ANOVA

There was a direct low statistically significant correlation between age and latency P100 for the right eye (*r*=0.311; *P* = 0.01) or latency P100 for the left eye (*r*=0.293; *P* = 0.049) (Table 2). UPDRS item 4 grade was directly correlated with latency P100 for the right eye (*r*=0. 0.449; *P* <0.001) and with latency P100 for the left eye (*r*=0. 378; *P* = 0.003) but not with the difference in latency between eyes (*P* = 0.1). No statistically significant correlation was observed between age and the difference in latency between eyes (*P* = 0.2). No statistically significant correlations were observed between age of onset, Hoehn and Yahr scale, UPDRS scale, MMSE scale, LARS scale, DAS scale, and latency P100 for the right eye, left eye, or difference in latency between eyes. There was a weak statistically significant correlation between anhedonia score SHAPS and latency P100 for the right eye (*r*=0.284; *P* = 0.02).

**Table 2:** Correlation between latency P100 for the right eye, left eye, or difference in latency between eyes and several scales and demographic variables

Variable	Latency P100 for the right eye		Latency P100 for the left eye		Difference in latency between eyes	
	*r*	*P*	*r*	*P*	*r*	*P*
Age	0.311	0.01^†^	0.293	0.05^†^	0.139	0.2^†^
Age at onset	0.166	0.2^†^	0.059	0.6^†^	0.082	0.5^†^
Hoehn and Yahr scale	0.215	0.1^††^	0.112	0.3	0.057	0.6^††^
UPDRS item 4 grade	0.449	<0.001^††^	0.378	0.003^††^	0.171	0.1^††^
UPDRS scale	0.228	0.08^†^	0.193	0.1^†^	0.029	0.8^†^
MMSE scale	0.056	0.6^†^	0.017	0.9^†^	0.143	0.2^†^
Apathy score LARS	0.030	0.8^†^	0.003	0.9^†^	0.095	0.4^†^
Apathy score DAS	0.112	0.4^†^	0.119	0.3^†^	0.106	0.4^†^
Anhedonia score SHAPS	0.284	0.02^†^	0.119	0.06^†^	0.025	0.8^†^

UPDRS, Unified Parkinson's Disease Rating Scale; MMSE, Mini-Mental State Examination; LARS, Lille apathy rating scale; DAS, Dimensional Apathy Scale; †Pearson correlation; ††Spearman rank correlation

## DISCUSSION

The investigation aimed to discern VEP latencies in patients with Parkinson's disease and their potential correlations with various clinical parameters. The observed symmetry in P100 latencies between the right and left eyes suggests a harmonious VEP response between ocular pathways, affirming the consistency in visual processing across both eyes. However, intriguingly, this uniformity contrasts with the significantly associated prolonged latencies in both eyes concerning higher UPDRS item 4 grades. This hints at a plausible connection between altered VEP responses and the severity of motor and non-motor symptoms in PD [2,14]. The study evaluated the influence of predominant parkinsonism types on latency differences between the eyes. Notably, patients with symmetrical parkinsonism displayed shorter latency differences between their eyes compared to those with unilateral predominance. This observation suggests a potential interrelation between asymmetry in motor symptoms and variations in VEP latencies. This nuanced association underscores the intricate dynamics between motor asymmetry and visual processing alterations, offering a novel perspective in understanding the pathophysiological mechanisms of PD [15,16]. One possible hypothesis is rooted in the complex network of neural circuits that govern both motor control and visual processing. Parkinson's disease is characterized by the degeneration of dopaminergic neurons, leading to disruptions in various brain regions, including the basal ganglia, which plays an important role in motor functions [17,18]. It is plausible that the noted shorter latency differences in symmetrical parkinsonism could be linked to a more balanced or symmetrical distribution of dopaminergic dysfunction across the basal ganglia and related circuits [19]. In contrast, unilateral predominance may result in a more pronounced asymmetry in dopaminergic deficits, impacting not only motor control but also visual processing pathways. This asymmetry in dopaminergic dysfunction could contribute to alterations in the processing of visual stimuli, reflected in prolonged latency differences between the eyes [20]. The complex interconnections between the basal ganglia and visual processing areas, such as the occipital cortex, may contribute to the observed variations in VEP latencies. Asymmetric dopaminergic deficits could lead to imbalances in the modulation of these circuits, affecting the timing of visual signal transmission and processing [21]. The correlation analysis underscored a moderate statistically significant association between age and P100 latency for both the right and left eyes. This suggests that advanced age might contribute to subtle changes in VEP responses, consistent with prior studies. However, no significant correlations were discerned between the age of onset, UPDRS scale, MMSE scale, LARS scale, DAS scale, and VEP latencies. Intriguingly, a low yet significant correlation emerged between anhedonia scores measured by SHAPS and the latency of P100 in the right eye. This intriguing association indicates a potential link between visual processing alterations and specific non-motor symptoms in PD, highlighting the complexity of symptomatology in this condition. The rehabilitation efforts and the improvement of the quality of life of subjects with PD can thus be profoundly impacted by the visual changes occurring in these patients, both directly and indirectly. Directly, these alterations will impair equilibrium and motor coordination, for which visual processing is crucial, making it more challenging to carry out regular tasks safely. Additionally, the success of rehabilitation therapies that rely on visual feedback, like certain physical therapy techniques, may be diminished, so therapists might need to adjust exercises by integrating other sensory inputs or offering extra guidance [22]. For these cases, vestibular rehabilitation has been investigated as a possible therapeutic strategy to help patients with visual impairments improve their balance and coordination. This approach might enhance the overall effectiveness of rehabilitation programs by reducing the impact that visual impairments have on balance and motor performance [23]. Indirectly, considering the correlation between certain changes in the P100 wave and the score obtained in the evaluation of anhedonia, visual changes may predict greater difficulty in maintaining an effective rehabilitation program for a PD patient with disturbances in the motivational sphere. Comparative analyses with existing literature validate certain aspects of our findings, demonstrating consistent associations between VEP alterations and disease severity markers in PD. However, discrepancies were observed concerning the correlations between VEP latencies and specific non-motor symptoms or cognitive measures, underscoring the multifaceted and heterogeneous nature of PD manifestations. These variations might stem from differences in sample characteristics, study methodologies, or the multifaceted nature of PD itself.

Acknowledging the study's limitations, including its cross-sectional design, relatively modest sample size, and potential unaccounted confounders, calls for cautious interpretations. Future investigations with larger cohorts and longitudinal designs could provide a more comprehensive understanding of the intricate associations between VEP latencies and the diverse spectrum of PD manifestations, shedding light on the underlying mechanisms driving these complex interactions. Investigating VEP latencies in the context of Parkinson's disease not only helps to clarify the neural basis of visual processing changes but also creates opportunities for potential diagnostic and therapeutic progressions. This comprehensive investigation paves the way for deeper explorations into the intricate relationships between visual dysfunction, motor symptoms, and non-motor manifestations in PD, fostering a more holistic approach to understanding this multifaceted neurodegenerative disorder.

## CONCLUSION

The electrophysiological changes discussed may reveal the extent of dopaminergic depletion, including at the retinal level. The investigation using VEPs in patients diagnosed with PD revealed intriguing patterns and correlations. However, it should be noted that unilateral predominance of parkinsonism is associated with more significant alterations in P100 latency differences compared to subjects with symmetric parkinsonism. Moreover, the increase in P100 wave latency, along with the increase in scores obtained when assessing other non-motor parameters, anhedonia (through SHAPS), and lack of initiative and motivation (through UPDRS item 4), illustrate the intricacy of damage at the level of the mechanisms involved in the non-motor sphere. Thus, these testing tools could be useful as part of the strategy for assessing the extent and trajectory of the disease as well as in establishing the most appropriate guidelines in the process of rehabilitation and the maintenance or improvement of the quality of life of PD patients.
